# Supramolecular assembly of coumarin 7 with sulfobutylether-β-cyclodextrin for biomolecular applications

**DOI:** 10.3389/fchem.2023.1245518

**Published:** 2023-09-05

**Authors:** T. C. Gayathry, Monika Gaur, Lopamudra Mishra, Monalisa Mishra, Nilotpal Barooah, Achikanath C. Bhasikuttan, Jyotirmayee Mohanty

**Affiliations:** ^1^ Radiation and Photochemistry Division, Bhabha Atomic Research Centre, Mumbai, India; ^2^ Homi Bhabha National Institute, Training School Complex, Mumbai, India; ^3^ Department of Life Science, National Institute of Technology Rourkela, Rourkela, India

**Keywords:** 6 host-guest complex, sulfobutylether-β-cyclodextrin, coumarin 7, photostability, stimuli-responsive behavior, bioimaging

## Abstract

Coumarins, in general, exhibit a wide range of photophysical characteristics and are highly sensitive to their microenvironment, and, therefore, their fluorescence characteristics have attracted immense attention as sensors in chemical and biological systems. In the present study, the supramolecular interaction of a bichromophoric coumarin dye, namely, Coumarin 7 (C7) with sulfobutylether-β-cyclodextrin (SBE_7_βCD) macrocyclic host at different pH conditions has been investigated by using optical spectroscopic techniques such as absorption, steady-state and time-resolved emissions, and circular dichroism measurements and compared with that of βCD. Considerable enhancement in the fluorescence intensity and lifetime of C7 on complexation with SBE_7_βCD proposes that non-radiative processes like TICT behavior are strictly hindered due to the confinement in the host cavity experienced by the C7 dye. The increase in the rotational correlation time evaluated from the fluorescence anisotropy decay kinetics further confirms the formation of tightly bound inclusion complexes. The binding constant values reveal that the monocationic form of dye at pH 3 shows ∼3 times stronger interaction with SBE_7_βCD than the neutral form of dye at pH 7 due to strong electrostatic cation-anion interaction. SBE_7_βCD:C7 exhibits an improved photostability and an upward p*K*_a_ shift of 0.4 unit compared to the contrasting downward p*K*_a_ shift of 0.5 with the βCD. The enhanced fluorescence yield and increased photostability have been exploited for bioimaging applications, and better images were captured by staining the *Drosophila* fly gut with the SBE_7_βCD:C7 complex. The enhancement in the binding interaction and the emission intensity were found to be responsive to external stimuli such as small competitive binders or metal ions and nearly quantitative dissociation of the complex was demonstrated to release the dye and would find stimuli-responsive applications.

## Graphical Abstract

**Figure:**
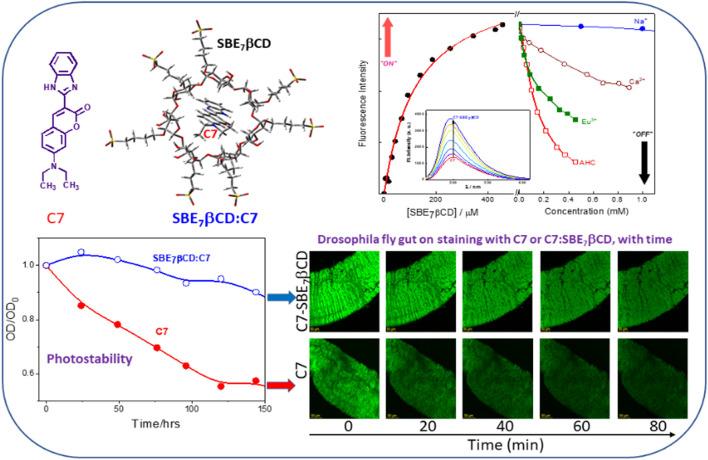
defect repair.

## Introduction

Supramolecular host-guest assembly of fluorescent dyes/drugs using macrocyclic receptors through non-covalent electrostatic and hydrophobic interactions is an area of considerable research interest as molecular properties of these dyes/drugs can be modulated adequately through these interactions (Bhasikuttan et al., 2011; Barooah et al., 2022). This is because the guest dye/drug experiences a totally different environment inside the host cavity compared to that in the bulk solution (Mohanty, and Nau, 2005; Bhasikuttan et al., 2011). Such molecular assemblies are of enormous importance for applications in various areas such as drug delivery, photodynamic therapy, organic electronics, sensors, fluorescent probes, and catalysts, (Khurana et al., 2019a; Khurana et al., 2019c; Ruz et al., 2021; Barooah et al., 2022; Siddharthan et al., 2023). Over the years, several molecular systems have been investigated to establish the usages of the non-covalently stabilized host–guest complexes using cavitand host molecules, namely, calixarenes, cyclodextrins, and cucurbiturils and their derivatives. (Khurana et al., 2019a; Khurana et al., 2019b; Mehra et al., 2019; Kadam et al., 2020; Barooah et al., 2022; Bhasikuttan et al., 2011; Siddharthan et al., 2023). Host-guest complexation can vary the emission yields, excited state relaxation pathways, and photostability of the guest molecules (Mohanty, and Nau, 2005). Due to this host-guest assembly formation, there is a possibility of de-aggregation of the dyes or drugs which will enhance the dye/drug solubility in the aqueous medium. In this context, we have chosen a bichromophoric coumarin derivative as a guest dye, namely, coumarin 7 (C7). The bichromophoric coumarin dye C7 consists of a benzimidazole moiety attached to a 7-N,N′-diethylaminocoumarin moiety. Coumarin dyes, in general, have obtained ample attention in various fields on account of their potential application and are among the few systems explored in-depth by photo-physicists (Jones et al., 1980; Jones et al., 1985). In addition to their widespread utility in dye laser systems, coumarin dyes also find their usage in bioimaging and other biological systems (Madhavan et al., 2003; Signore et al., 2010; Chandrasekaran et al., 2014; Chandrasekaran et al., 2015). Some of these dyes such as coumarin 6 (C6), coumarin 7 (C7), and coumarin 30 (C30) find specific usages in organic light-emitting diodes (OLEDs) (Swanson et al., 2003; Lee et al., 2004) in addition to being popular fluorescent probes for evaluating micro-environmental polarity changes (Klymchenko, and Demchenko, 2002; Vasylevska et al., 2007; Wagner, 2009; Das et al., 2021). The introduction of a benzimidazole moiety at the 3-position on the coumarin core allows extended conjugation among the two units and also creates a protonation site, which can significantly change the optical behavior of these dyes. In earlier work, we have established such supramolecular p*K*_a_ shift due to host-guest interaction in C7 (Δp*K*_a_ = 4.6) and C30 (Δp*K*_a_ = 3.0) through the interaction of appropriate synthetic receptors such as cucurbit [7] uril (CB7) (Barooah et al., 2014).

**SCHEME 1:**
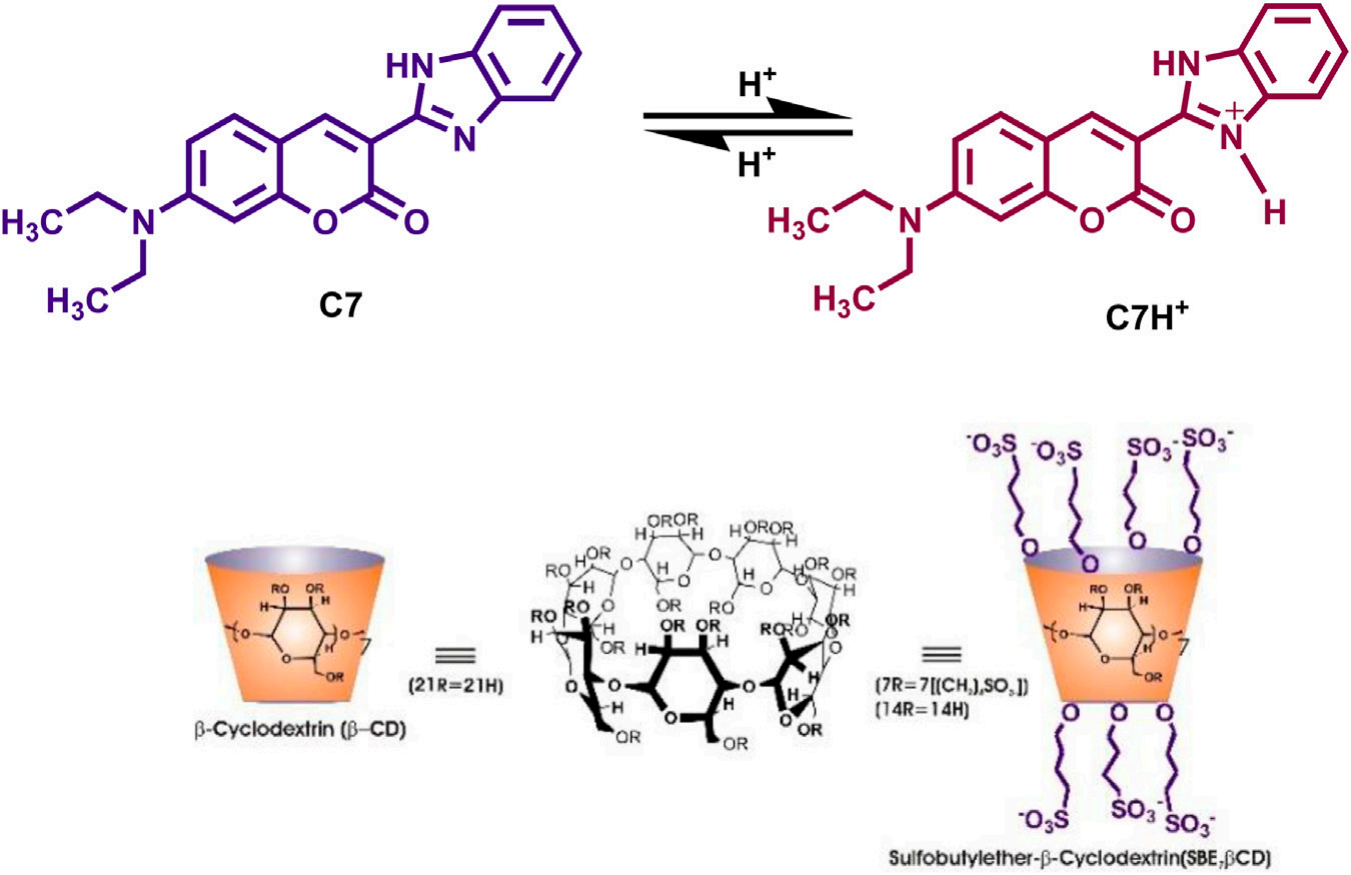
Chemical structures of Coumarin-7 (C7), *ß*-cyclodextrin (βCD), and sulfobutylether-β-cyclodextrin (SBE_7_βCD).

In the present work, we have employed the non-covalent host-guest interaction of sulfobutylether-β-cyclodextrin (SBE_7_βCD, Scheme 1) to modulate the photophysical properties and the photostability of coumarin 7. SBE_7_βCD, a water-soluble *ß*-cyclodextrin (βCD) derivative, presents derivatized portals having several hydroxyl and sulphonate groups that extend the effect of host hydrophobic cavity (Loftsson and Brewster, 1996; Kale et al., 2005; Jain et al., 2011). SBE_7_βCD is synthesized by derivatizing the βCD hydroxyl groups with sulfobutyl groups (Loftsson and Brewster, 1996; Kale et al., 2005; Jain et al., 2011). The extended SBE_7_βCD portals with sulphonate groups can uptake appropriate guests through electrostatic interactions. Moreover, the SBE_7_βCD has added advantages of increased aqueous solubility, improved interaction with drugs, and low toxicity (Loftsson, and Brewster, 1996; Kale et al., 2005; Jain et al., 2011). Also, SBE_7_βCD exhibits much higher hemocompatibility compared to the βCD (Das et al., 2019). We have shown that SBE_7_βCD is an efficient inhibiter of fibril formation and effectively disintegrates the mature fibrils into nontoxic small particles (Shinde et al., 2017). In our earlier studies, the improved complexation behavior of SBE_7_βCD toward 4’,6-diamidino-2-phenylindole (DAPI), a well-known fluorescent probe for DNA, and its application toward stimuli-responsive on-off switches (Shinde et al., 2015), a fluorescence-based sensor for a biogenic amine, tyramine (Khurana et al., 2019b) and water-based dye laser system using rhodamines have been established (Khurana et al., 2018). In one of the recent studies, we have shown supramolecular nanorods of 5,10,15,20-tetrakis (4-*N*-methylpyridyl)porphyrin dye/drug with SBE_7_βCD and demonstrated them as a superior antibacterial/antitumor agent apart from being an effective photosensitizer (Khurana et al., 2019c). Of late, SBE_7_βCD has also been used to enhance the antibacterial activity of a drug supplement, sanguinarine (Kadam et al., 2020). Herein, the supramolecular assembly formation of coumarin 7 (C7) with SBE_7_βCD has been investigated. The assembly formation significantly modulates/improves the fluorescence behavior and photostability of C7 which have been explored for the bio-imaging application using the *Drosophila* fly model.

## Materials and methods

### Materials

Sulfobutylether-β-cyclodextrin sodium salt, having 6.4 degrees of substitution, was obtained from AdventChemBio Pvt. Ltd., India, and used without further purification. The coumarin 7 was obtained from Aldrich. Nanopure water obtained from a Millipore Elix 3/A10 water purification system (conductivity less than 0.1 μS cm^−1^) was used to prepare the experimental solutions.

### Spectroscopic and bio-imaging methods

Absorption and emission spectra were obtained using a UV-Vis spectrophotometer (model 3,600 plus) from Shimadzu and a spectrofluorometer (FS5, Edinburgh Instruments), respectively. Fluorescence quantum yield (Φ_f_) of the SBE_7_βCD:C7 assembly was estimated from the area under the curve in comparison with that of free C7H^+^ in water (Φ_f_ (pH 3) = 0.05) (Barooah et al., 2014). Time-resolved fluorescence and fluorescence anisotropy measurements were carried out using a TCSPC (time-correlated single photon counting) spectrometer (Horiba JobinYvon, United Kingdom). For details, see Supplementary Material. ^1^H NMR measurements were carried out using a Bruker Avance WB spectrometer (800 MHz) at Tata Institute of Fundamental Research (TIFR), Mumbai, India. Circular dichroism (CD) data measurements were performed using a BioLogic spectrometer (MOS-500). The spectra were measured in the wavelength range of 300–650 nm using a quartz cuvette (1 cm path length). The details of the photostability and bioimaging methods have been provided in the Supplementary Material.

## Results and discussion

### Interaction of C7 with SBE_7_βCD; absorption and emission features

Since the C7 dye structure contains protonatable nitrogens, it is expected that the solution pH would be of concern when the physicochemical properties are measured in an aqueous medium. In this context, the pK_a_ of C7 has been estimated (aqueous solution containing ∼0.2% ethanol for solubility) from the absorption spectral changes with pH, and the results are shown in Supplementary Figure S1. The absorption shows a spectral maximum at 475 nm at pH ∼3 (Barooah et al., 2014), and on a gradual increase in the solution pH, the spectral maximum shifts to a lower wavelength region. The hypsochromic shift with increasing pH indicates the absorption changes due to the protonation-deprotonation equilibrium of C7 in the pH range studied. The pH-dependent absorbance changes were plotted and analyzed (Supplementary Figure S1) following the relation Eq. 1 (Jadhav et al., 2015).

$$A_{obs}=\frac{A_{{DyeH}^{+}}^{\infty }}{\left\{1+10^{pH-pK_{a}}\right\}}+\frac{A_{Dye}^{\infty }}{\left\{1+10^{{pK}_{a}-pH}\right\}}$$ (1)

Here, A_obs_ represents the absorbance value at any pH, $A_{Dye^{+}}^{\infty }$ and $A_{Dye}^{\infty }$ are the maximum expected absorbance values of the DyeH^+^ (**C7H** ^**+**^ at pH 3) and Dye (**C7** at pH 7) forms, respectively. From the analysis of the pH curve (inset Supplementary Figure S1), the p*K*_a_ value was estimated as 5.03 ± 0.08 and is in close agreement with the value of 5.12 reported before (Barooah et al., 2014).

**FIGURE 1:**
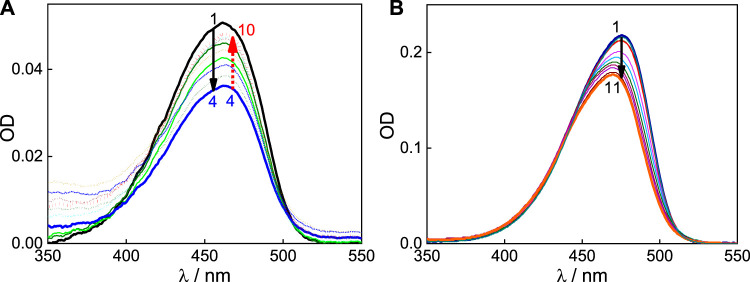
**(A)** Absorption spectra of C7 (pH 7) with [SBE_7_βCD]/μM: 0 (1); 5 (2); 10 (3); 17.5 (4); 30 (5); 37.5 (6); 62.5 (7); 138.5 (8); 187.5 (9) and 262.5 (10). **(B)** Absorption spectra of C7 at pH 3 with [SBE_7_βCD]/µM: 0 (1); 0.5 (2); 2 (3); 5 (4); 10 (5); 20 (6); 39.0 (7); 67.6 (8); 104.0 (9); 143.8 (10); 181.8 (11).

An aqueous solution of C7 exhibits a broad absorption profile centered at 462 nm (Figure 1 and Supplementary Figure S1), and in an acidic medium, the spectral profile gets narrower and the absorption maximum shifts to a higher wavelength, 475 nm (Barooah et al., 2014). Since the evaluated p*K*_a_ is ∼5, it is considered that at a pH below four, C7 mostly exists as monocationic (C7H^+^), and at a pH above six, C7 exists in its neutral form (C7). Therefore, the interaction C7H^+^ and C7 forms with the SBE_7_βCD host was investigated at pH 3 and pH 7, respectively. At pH ∼7, with increasing concentration of SBE_7_βCD up to ∼17.5 μM, the absorbance of C7 at 462 nm decreases and beyond this concentration of SBE_7_βCD, the absorbance increases and attains saturation with ∼260 μM of SBE_7_βCD (Figure 1A). These absorption spectral changes with SBE_7_βCD points to a two-stage interaction of SBE_7_βCD with C7 dye. At pH 3, the interaction of SBE_7_βCD with C7H^+^ shows a gradual blue shift from 475 nm to 469 nm along with a hypochromic shift (Figure 1B). All these absorption spectral changes in C7 on interaction with SBE_7_βCD at both the pH conditions suggest the complex formation between C7 dye and SBE_7_βCD host. The hypochromic effect indicates the lower extinction coefficient of SBE_7_βCD-C7/C7H^+^ complexes than C7/C7H^+^ respectively. This is due to the change in the transition probability of C7/C7H^+^ on their encapsulation in the macrocyclic cavity of SBE_7_βCD.

**FIGURE 2:**
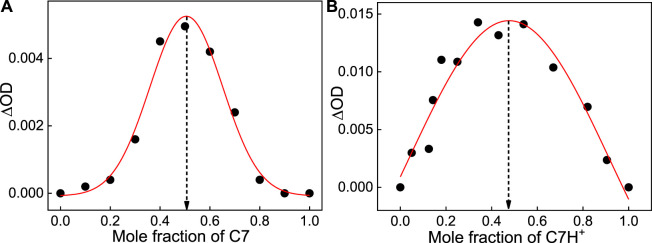
Jobs plots of C7:SBE_7_βCD system at pH ∼7 **(A)** and pH ∼3 **(B)**.

To evaluate the stoichiometric composition of the host-guest complexes, Jobs plot measurements, employing the continuous variation method, were carried out at both pH conditions. The overall concentrations of the guest dye and SBE_7_βCD host are kept constant. The changes in the absorbance with the mole fraction of dye/host has been plotted. As displayed in Figures 2A, B, the Jobs plots for both the complexes at pH 7 and 3 show the inflection point at a mole fraction of ∼0.5, representing 1:1 stoichiometry for the SBE_7_βCD:C7/C7H^+^ complexes.

**FIGURE 3:**
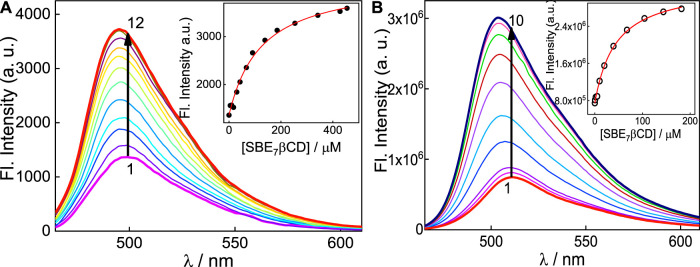
**(A)** Fluorescence spectra of C7 at pH 7 with [SBE_7_βCD]/μM: 0 (1); 10 (2); 30 (3); 42.5 (4); 67.0 (5); 90.8 (6); 138.5 (7); 185.4 (8); 254 (9); 342 (10); 427.5 (11) and 454.5 (12). *λ*_ex_ = 445 nm, *λ*_mon_ = 500 nm. **(B)** Fluorescence spectra of C7 at pH 3 with [SBE_7_βCD]/μM:0 (1); 0.5 (2); 2.0 (3); 5 (4); 10.0 (5); 20.0 (6); 39.2 (7); 67.6 (8); 104.2 (9); 143.8 (10); 181.8 (11). *λ*_ex_ = 455 nm, *λ*_mon_ = 510 nm. Insets show the binding isotherms of the SBE_7_βCD:C7 system at respective pH 7 and pH 3.

It has been reported that N,N′-dialkyl substituted coumarins are weakly emissive in water (Dahiya et al., 2005). In these structures, electronic conjugation is more feasible and results in more planar intramolecular charge transfer (ICT) states in the ground state, which in the excited state gets converted to a non-emissive twisted intramolecular charge transfer (TICT) state, which is more prevalent in water (Dahiya et al., 2005). C7 exhibits moderately intense emission (Φ_f(C7, pH7)_ = 0.15) in aqueous solution at pH 7 (Barooah et al., 2014), with the emission maximum at around 500 nm (Figure 3A). To this, incremental addition of SBE_7_βCD provided steady enhancement in emission and a gradual blue shift of the spectral maximum from 500 nm to 495 nm, which eventually saturates with the addition of ∼455 μM of SBE_7_βCD (Figure 3A). The quantum yield of C7 increases by two folds from 0.15 to 0.3 (Table 1) in the presence of saturated concentration of SBE_7_βCD. Instead, C7H^+^ (at pH 3) dye shows very weak emission intensity in aqueous solution (Φ_f(C7)_ = 0.05) (Barooah et al., 2014), and the addition of SBE_7_βCD brings out an increase in the emission intensity (quantum yield increases from 0.05 to 0.13 (Table 1)) and the emission maximum blue shifted from 512 nm to 503 nm. These changes attain completion with ∼182 μM SBE_7_βCD (Figure 3B). The increased emission intensity observed for both the C7/C7H^+^forms with SBE_7_βCD is ascribed to the inclusion complexes formed, where the coumarin probe is placed inside the hydrophobic cavity of SBE_7_βCD. The blue shift in band maximum arises as the energy gap between the ground and excited state increases in the nonpolar cavity. As observed, the C7H^+^ form is weakly emissive in the aqueous medium, which is understood as the case of the interplay of ICT and TICT states and is discussed in the later part.

**TABLE 1:** Photophysical parameters of C7 Dye with SBE_7_βCD at different pH conditions in aqueous solutions. *λ*_ex_ = 445 nm and $\lambda_{em}^{mon}$ = 510 nm.

Dye	[SBE_7_βCD] (mM)	$\lambda_{abs}^{\max}$ (nm)	$\lambda_{em}^{\max}$ (nm)	ϕ_f_	τ_1_ (ns) (% a_1_)	τ_2_ (ns) (% a_2_)	χ^a^	^b^<τ> (ns)	^c^τ_r_ (ns)
C7 (pH 7)	0.0	450	500	0.15	0.19 7)	1.15 (93)	1.0	1.08	0.24 ± 0.03
		^c^462							
C7 (pH 7)	2.0	448	495	0.30	1.09 (14)	2.67 (86)	1.1	2.45	0.89 ± 0.05
		^c^462							
C7H^+^ (pH 3)	0.0	475	512	0.05	0.16 (96)	1.94 4)	^d^1.3	0.23	---
C7H^+^ (pH 3)	0.2	469	503	0.13	0.22 (22)	2.18 (78)	^d^1.4	1.75	1.02 ± 0.05

^c^ Absorption maximum of C7 in tris buffer (10 mM, pH 7.4).

^b^ The average lifetime value is associated with 5% uncertainties.

^d^ Slightly higher χ

^a^ Value due to the low intensity and fast decay profile at pH 3.

The particulars of the host-guest interactions were evaluated from the fluorescence enhancement, and the insets of Figure 3 display the fluorescence titration curves obtained for both forms of C7 dye in the presence of SBE_7_βCD. For the system at pH 3, the binding constant (*K*) for the C7H^+^ with SBE_7_βCD host (H) was estimated by assuming a 1:1 stoichiometry. Here, the fluorescence intensity observed, I_f_, accounts for the total of the emission intensities from the free dye and H:Dye and would represent their respective concentrations in the system. Therefore,

$$I_{f}=I_{f}^{0}\frac{{\left[Dye\right]}_{eq}}{{\left[Dye\right]}_{0}}+I_{H:Dye}\frac{{\left[H:Dye\right]}_{eq}}{{\left[Dye\right]}_{0}}$$ (2)

where I_f_^0^ is the intensity without the presence of the host and I_H:Dye_ represents intensity when 1:1 host-guest complexation is saturated. [Dye]_0_ and [H]_0_ indicate the total concentrations of Dye and H used. Eq. 2 can be rearranged into Eq. 3 (Dutta Choudhury et al., 2009; Siddharthan et al., 2023) as a modified Benesi-Hildebrand relation,

$$I_{f}=\frac{I_{f}^{0}+I_{H:Dye}K{\left[H\right]}_{0}}{1+K{\left[H\right]}_{0}}$$ (3)

From the binding curves (Figure 3 insets), the binding constant value is estimated by using Eq. 3 is (2.3 ± 0.2) ×10^4^ M^-1^ for SBE_7_βCD:C7H^+^ complex at pH 3 and (8.1 ± 0.8) ×10^3^ M^-1^ for SBE_7_βCD:C7 at pH 7). This result specifies that the monocationic form of C7H^+^ at pH 3 shows ∼3-fold stronger interaction with SBE_7_βCD than the interaction of neutral C7 with SBE_7_βCD at pH 7. Since the binding constant values of both the forms of C7 with SBE_7_βCD are higher than the reported binding constant value (1.09 ×10^2^ M^-1^) of *ß*-CD:C7 (Chandrasekaran et al., 2015), it is presumed that the interaction between them is mainly driven through electrostatic interaction, anion at the portals with the cation of C7H^+^, or through ion-dipole interaction involving the anion at the portals with the induced dipole due to the ICT character of the C7, along with the hydrophobic interaction of the core βCD cavity.

**FIGURE 4:**
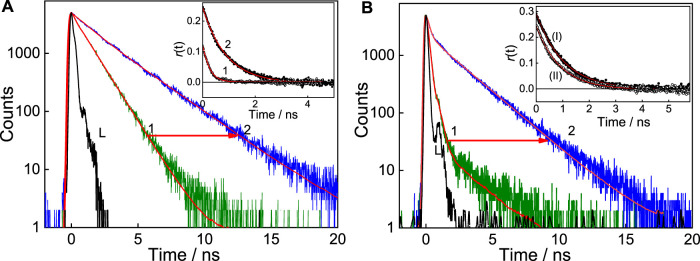
**(A)** Fluorescence decay of C7 in the absence (1) and presence of ∼454 μM SBE_7_βCD (2) at pH 7. **(B)** Fluorescence decay of C7 in the absence (1) and presence of 181 μM SBE_7_βCD (2) at pH 3, *λ*_ex_ = 445 nm and *λ*_em_ = 510 nm. Inset **(A)** shows the fluorescence anisotropy decay of C7 in water in the absence (1) and presence of SBE_7_βCD (454 µM) at pH 7, *λ*_ex_ = 445 nm. *λ*_em_ = 530 nm. Inset **(B)** shows the anisotropy decay of C7 in the presence of SBE_7_βCD (181 µM) at pH 3 (I) compared with pH 7 (II), *λ*_ex_ = 445 nm. *λ*_em_ = 530 nm.

The excited state decay traces for each set of experiments were carried out at a specific pH solution. At pH 7, free C7 dye displays biexponential decay kinetics providing a short lifetime component of 0.19 ns having a minor contribution (7%) and a longer lifetime component, i.e., 1.15 ns with an approximately 93% contribution (Figure 4A, trace 1), and the average lifetime is estimated as 1.08 ns. With the addition of ∼2 mM SBE_7_βCD, the short lifetime increases from 0.19 to 1.09 ns with a slight increase in the contribution from 7% to 14%, and the long component also increases to 2.67 ns and the average lifetime increases to 2.45 ns (Table 1). Whereas at pH 3, the decay trace of C7 also follows biexponential fitting with a very short lifetime of 0.17 ns having 96% contribution and a long lifetime of 1.94 ns with a negligible contribution (Figure 4B, trace 1). After the addition of saturated concentration (∼200 μM) of SBE_7_βCD to the C7 solution, the long lifetime increases to 2.18 ns with increased contribution, and the short lifetime increases slightly (Figure 4B, trace 2). The average lifetime increases from 0.23 ns to 1.75 ns (Table 1). Previous studies report that certain coumarin derivatives in polar/protic solvents display very high nonradiative decay rates (Jones et al., 1980; Jones et al., 1985; Barik et al., 2005). Such fast excited-state relaxation processes mostly happen due to the favorable ICT to TICT conversion viable in polar protic solvents (Satpati et al., 2009). Factors that bring constraints on such intramolecular motions will prevent the population of the TICT state and thus enhance the probability of radiative emission (Barooah et al., 2014). In general coumarin dyes displaying faster decay are understood to originate from an interplay of the charge ICT state and the restricted TICT state. The neutral form of C7 exists in a partial charge-separated intramolecular charge transfer state adopting a near planar conformation. Whereas, on protonation, the delocalization of nonbonding electrons of the diethyl amine group is restricted and the planar structure gets distorted to a twisted intramolecular charge transfer (TICT) state, which corresponds to a favorable geometry for the enhanced nonradiative decay and lower fluorescence yield (Jones et al., 1985; Satpati et al., 2009). On the other hand, excited state proton transfer/hydrogen bonding effects are seen prominently in the excited state decay of benzimidazole moieties as such interactions contribute to a faster decay (Shaikh et al., 2009). Further, since the polarity of the microenvironment inside the SBE_7_βCD cavity is expected to be lower than the bulk water which may also contribute to the lifetime parameter. In the complexed state, for both C7 and C7H^+^, all these nonradiative channels are hindered due to their encapsulation/protection in the SBE_7_βCD cavity and are the reason for the increase in the excited-state lifetimes (Ahmed et al., 2017) of C7 in the presence of SBE_7_βCD at both pH 7 and pH 3, presented in Figure 4.

### Fluorescence anisotropy study of C7 with SBE_7_βCD

Time-resolved fluorescence anisotropy, *r*(t), provides the extent of polarization of the emission with time (Lakowicz, 2006). Measurement of fluorescence anisotropy decay provides rotational correlation time (τ_r_) which correlates to the size of the emitting fluorophore. This methodology is used to evaluate the change in the size of the fluorophore, thereby confirming complexation and stoichiometry. As per the Stokes-Einstein relationship, τ_r_ for the complex can be related to its rotational diffusion coefficient and the viscosity of the environment by Eq. 4.

$$\tau_{r}=1\;/\;\left(6D_{r}\right),where\;D_{r}=RT/6V\eta$$ (4)

here, V represents the hydrodynamic molecular volume of the complex, η the medium viscosity, and T the absolute temperature. On complexation, it is expected that the rotational correlation time of the complex will increase compared to that of the free dye. We have carried out the time-resolved fluorescence anisotropy measurements of C7 dye in aqueous solution in the absence and presence of a saturated concentration of SBE_7_βCD at pH 7 and pH 3. The fluorescence anisotropy decays thus obtained for C7 with and without SBE_7_βCD at both the pH values are shown in the inset of Figure 4. The rotational correlation time (τ_r_) of free C7 at pH 7 obtained from the trace 1 (inset, Figure 4A) is ∼240 ps. Upon the addition of 454 μM of SBE_7_βCD, the τ_r_ value for the complex increases to ∼900 ps (trace 2, inset of Figure 4A). At pH 3, the τ_r_ value for the complexed C7 is approximately 972 ps. As discussed, the significant increase in the t_r_ in the complexed systems points out the inclusion of complex formation between coumarin dye with SBE_7_βCD at both pH conditions.

### ^1^H NMR measurements

**FIGURE 5:**
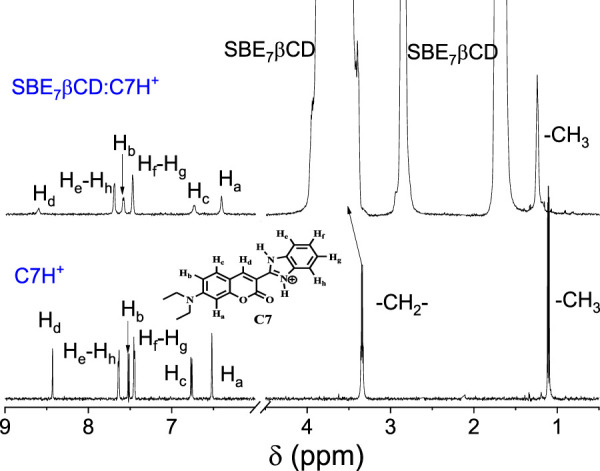
^1^H NMR spectra of C7H^+^ in the absence and presence of SBE_7_βCD in D_2_O at pD 3.

^1^H NMR studies were performed to get details about the binding sites of C7 with SBE_7_βCD in D_2_O at pD 3, as C7 at a neutral pH displays very low solubility. As shown in Figure 5, in the absence of SBE_7_βCD, the aromatic ring protons of the coumarin moiety, e.g., H_d_, H_b_, H_c_, and H_a_ appear at δ8.43s), 7.52 (d, *J* = 8 Hz), 6.77 (d, *J* = 8 Hz), and 6.52s), respectively along with the benzimidazole protons appearing as sets of doublets at δ7.64 (*J* = 8 Hz) and 7.45 (*J* = 8 Hz). The >CH_2_ and CH_3_ protons of the N,N′-diethylamino substituent appears at 3.35(q) and 1.11(t). The addition of SBE_7_βCD leads to a significant shift and broadening of all the aromatic protons of C7. In the presence of SBE_7_βCD, the coumarin protons H_d_ and H_b_ displayed a downfield shift to δ8.63 and 7.59, whereas the H_c_ and H_a_ protons showed an upfield shift to δ6.74 and 6.41. The benzimidazole protons and the CH_3_ and >CH_2_ protons displayed marginal downfield shift to δ7.70, 7.48, 3.42, and 1.25, respectively. As the SBE_7_βCD consists of an extended βCD cavity with the sulfobutylether arms at both portals, the length of the SBE_7_βCD cavity is long enough to accommodate the C7 dye vertically. Since the extended arms are not so rigid, the interaction offered by these side chains can be different, depending on the dye structure and its charge distribution and, hence, varying binding interactions.

### Geometry optimization studies

**FIGURE 6:**
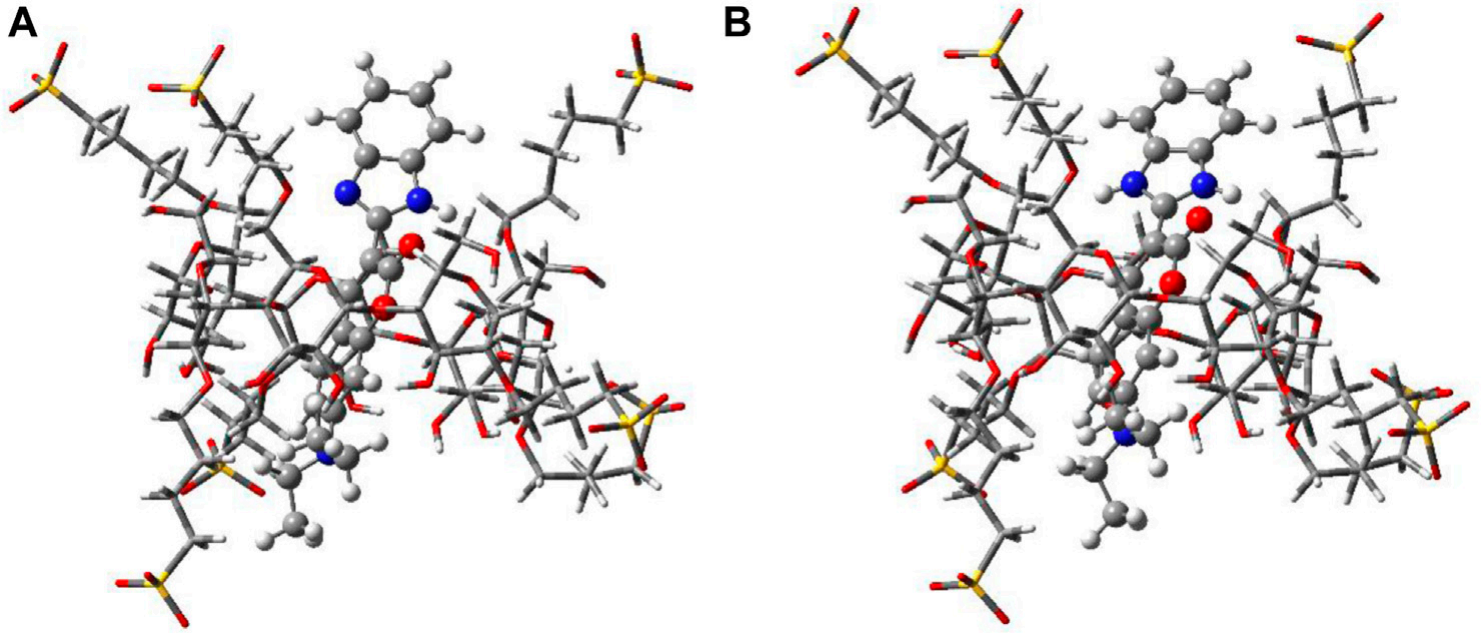
Geometry optimized structures of SBE_7_βCD:C7 **(A)** and SBE_7_βCD:C7H^+^ **(B)** complexes.

The geometry-optimized structures of the complexes in the ground state of both the protonated and neutral forms in the presence of SBE_7_βCD with the highest stabilization energy have been determined at the PM3 level by incorporating the molecular mechanics (MM) correction, using the Gaussian package (Figure 6) (Frisch et al., 1992). Here, optimization is done using several input geometries without any symmetry restraint, and solvent molecules are not considered for optimization. Among the geometries, the lowest ΔH_f_ values obtained for SBE_7_βCD:C7 (Figure 6A) and SBE_7_βCD:C7H^+^ (Figure 6B) complexes are −304.2 kcal/mol and −478.3 kcal/mol, respectively. In both complexes, the dye is positioned vertically through the center of the SBE_7_βCD cavity. However, in the case of the SBE_7_βCD:C7H^+^ complex (Figure 6B), the positive charge of the imidazole group comes close to the SO_3_^−^ groups of SBE_7_βCD, and, hence, the benzimidazole moiety remains slightly tilted with respect to the main coumarin moiety. In this arrangement, the SO_3_^−^ oxygen atoms are involved in strong H-bonding interactions with the ≥NH^+^ hydrogen as close as ∼1.7 Ả (Figure 6B). A larger ΔH_f_ value for SBE_7_βCD:C7H^+^ complex than the SBE_7_βCD:C7 complex indicates the better stabilization of the protonated benzimidazole moiety of C7H^+^ by the sulfonated groups of SBE_7_βCD through electrostatic interactions than the neutral form.

### Supramolecularly induced pK_a_ shift of C7 with SBE_7_βCD

**FIGURE 7:**
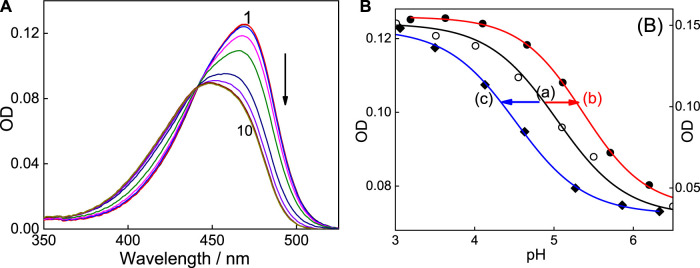
Absorption spectra of C7 in the presence of 2 mM SBE_7_βCD by varying pH of the solutions. pH values: 1) 3.2, 2) 3.6, 3) 4.0, 4) 4.6, 5) 5.0, 6) 5.6, 7) 6.1, 8) 6.7, 9) 7.2 and 10) 7.6. p*K*_a_ curve (variation of OD at 469 nm with pH of the solution) of C7 **(A)**, SBE_7_βCD:C7 complex **(B)** and βCD:C7 complex **(C)**.

**SCHEME 2:**
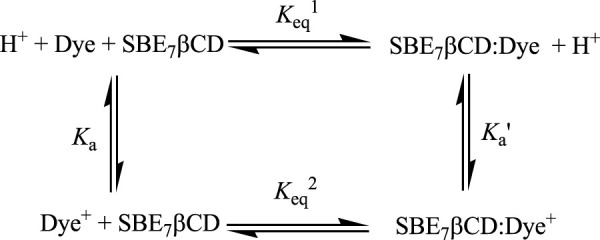
Four-state thermodynamic model.

Significant modulations are often observed in the protolytic equilibrium of the encapsulated guests due to the greater interaction of one of the forms with the host (Mohanty et al., 2006; Barooah et al., 2014; Shaikh et al., 2009; Barooah et al., 2012; Mehra et al., 2019; Siddharthan et al., 2023). Upward pK_a_ shifts are typically noticed for weak basic probes and cation-receptor/hydrogen bond acceptor hosts, whereas downward pK_a_ shifts are common for anion-receptor/hydrogen bond donor hosts. On this basis, the complexation consequence of SBE_7_βCD on the protolytic features of C7 dye has been studied by monitoring the variations in the absorption spectral profile of the dye with SBE_7_βCD (∼2 mM) with changes in pH, and the changes are plotted as shown in Figure 7. Considering the interaction of both forms of the dye with SBE_7_βCD, it is expected that the protolytic equilibria of the dye can be represented as a four-state model (Scheme 2) (Mohanty et al., 2006). *K*_a_ and $K_{a}^{\prime }$ denote the acid dissociation constants for the uncomplexed and complexed dye, respectively, and $K_{eq}^{1}$ and $K_{eq}^{2}$ designate the binding constants for the cationic (Dye^+^) and neutral (Dye) forms of the dye with SBE_7_βCD.

Experimentally, in the case of the C7:SBE_7_βCD complex, the absorption spectrum shows a peak position at ∼469 nm at pH ∼3. It is seen that the absorption spectrum shows a hypochromic blue shift with a peak position of around 450 nm and the spectrum becomes broad (Figure 7) with an increase in the pH of the solution. The curve (b) of the inset of Figure 7 shows the characteristics p*K*_a_ titration curve generated by plotting the changes in the absorbance with pH of SBE_7_βCD:C7 complex.

The evaluated p*K*_a_ value of the SBE_7_βCD-C7 complex is 5.4 ± 0.1, which established a supramolecular upward p*K*_a_ shift of ∼0.4 from that of C7 alone [curve (b) of the inset of Figure 7]. In another measurement, for comparison, the pK_a_ of C7 in the presence of native βCD was also evaluated. As presented in Figure 7 [curve (c)], the value turned out to be ∼4.5, which is a downward shift from the C7 value of p*K*_a_ 5.

All the complexation interaction of C7 dye has also been verified by following the changes in the circular dichroism spectra, which reveal the geometrical changes in the complex due to C7 and C7H^+^. This part is addressed in the Supplementary Materials along with a comparison of the SBE_7_βCD data with that of parent βCD.

### Photostability of C7 in the presence of SBE_7_βCD

**FIGURE 8:**
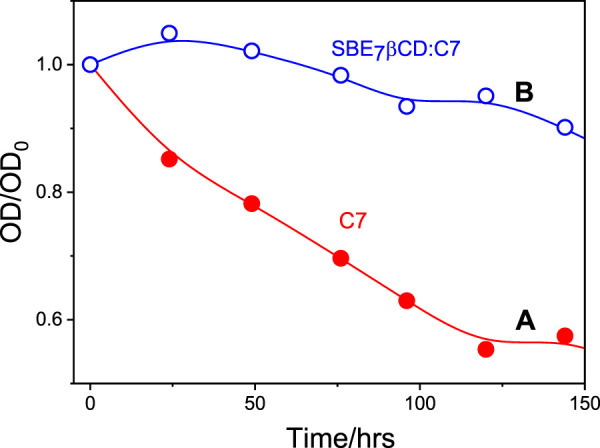
The absorbance changes of C7 at the absorption maxima with respect to their initial absorbance with time in the absence **(A)** and presence **(B)** of SBE_7_βCD (1 mM) at ambient conditions at pH 7.4.

It is quite common that the photostability of the guests (dyes and drugs) improves considerably due to the encapsulation by the macrocyclic hosts, where the dye/drug gets stabilized/protected in their hydrophobic cavity (Mohanty and Nau, 2005; El-Sheshtawy et al., 2018; Kadam et al., 2020; Siddharthan et al., 2023). In line with this, the photostability of C7 was compared with that of the C7- SBE_7_βCD complex at ambient conditions. The change in absorbance at the respective absorption maxima of the C7- SBE_7_βCD and C7 at pH 7.4 on exposure to daylight was monitored at different time intervals and the plots are presented in Figure 8. While C7 displayed faster degradation, the SBE_7_βCD:C7 complex displayed improved stability to ambient light exposure.

### Bio-imaging

**FIGURE 9:**
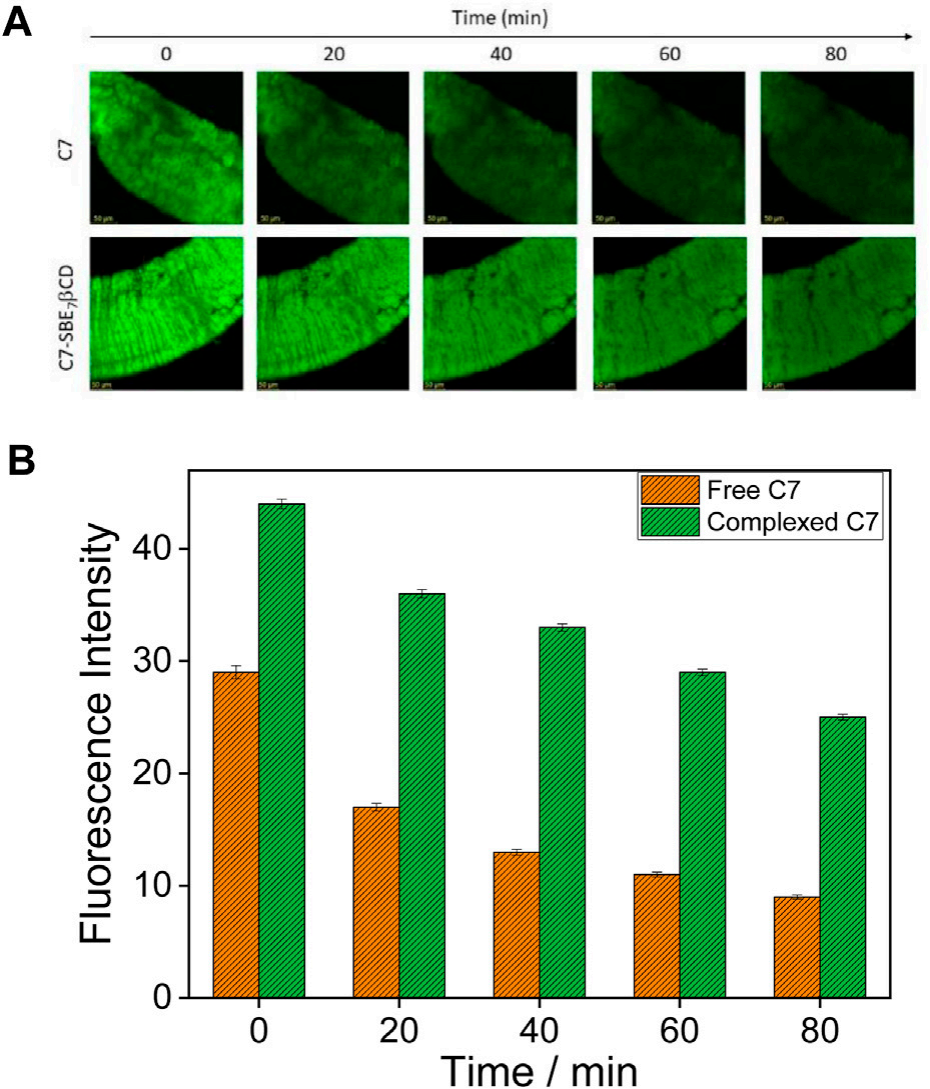
**(A)** Confocal images of the gut of the *Drosophila* fly which was incubated with 0.5 µM of C7 dye alone (upper panel) and with the SBE_7_βCD:C7complex (lower panel). The scale bar is 50 µm. **(B)** Quantification of fluorescence intensity of dye alone (orange bars) and dye in complex with SBE_7_βCDhost (green bars). *p-*values were calculated using two-sample t-tests: ***p < 0.0001.

The enhanced fluorescence intensity and photostability of C7 observed in the presence of SBE_7_βCD at pH 7.4 have been explored for the bioimaging study using the gut of the *Drosophila* fly model at different time intervals. For this, the gut of *Drosophila* was incubated with 0.5 µM of C7 alone, and the C7:SBE_7_βCD complex and images were taken in a time interval of 20 min using a confocal microscope (Figure 9). The gut stained with the C7:SBE_7_βCD complex displays higher brightness than the free C7. It is observed that the brightness of the gut stained with free C7 dye decreases largely compared to the brightness of the gut stained with the C7:SBE_7_βCD complex and is as shown in Figure 9A. The extent of reduction in the emission intensity of the C7-stained gut is much more (∼69%) compared to that of the complex-stained gut (∼43%) and the values are compared in Figure 9B.

### Stimuli-responsive tuning in the photophysical properties

Modulating and controlling the binding and release of small molecules, especially those which have purposeful roles in metabolic/biological processes, have received immense application in photodynamic therapy, drug delivery, and sensor applications (Dutta Choudhury et al., 2010; Khurana et al., 2019a; Barooah et al., 2022; Siddharthan et al., 2023). Since the host-guest complexes are formed by weak/reversible non-covalent interactions, these complexes respond to external stimuli such as pH, light, temperature, and metal ions, (Dutta Choudhury et al., 2009; Dutta Choudhury et al., 2010; Khurana et al., 2019b; Barooah et al., 2022; Siddharthan et al., 2023). In this regard, the stimuli-responsive behavior of SBE_7_βCD:C7 at pH 3 has been carried out by using chemical stimuli such as amantadine hydrochloride (AHC) and monovalent to trivalent metal ions as competitive binders to tune the absorption and fluorescence behavior.

By gradually adding AHC to the SBE_7_βCD:C7H^+^ complex, the fluorescence intensity decreases and attains saturation with ∼450 μM concentration of AHC, and the absorption and fluorescence spectral profiles revert back toward the free dye (Supplementary Figure S3). Considering that the complexation between SBE_7_βCD and C7H^+^ is mostly through Coulombic interactions with the SBE_7_βCD sulfonate portals, the outcome points to a competitive interaction of AHC and the dye toward the portals of SBE_7_βCD. This competitive binding interaction eventually replaces the bound dye and ruptures the SBE_7_βCD:C7H^+^complex. The breakage of the SBE_7_βCD:C7H^+^ complex in response to AHC stimulus is evident from Supplementary Figure S3.

**FIGURE 10:**
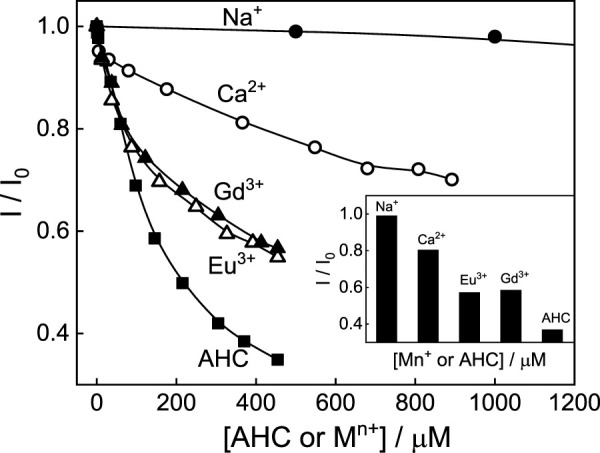
Normalized fluorescence intensity of SBE_7_βCD:C7H^+^complex with varying concentrations of competitive binders such as amantadine hydrochloride (AHC) or metal ions. Inset shows the effective fluorescence quenching at 400 μM of the competitive binder.

The competitive binding of monovalent to multivalent metal ions with the dye toward SBE_7_βCD has also been investigated. It is seen that by adding monovalent to trivalent metal ions to the SBE_7_βCD:C7H^+^ complex, the fluorescence intensity of C7H^+^ decreases and attains saturation with ∼90 mM of monovalent Na^+^ ion (Supplementary Figure S4) and ∼900 μM of bivalent Ca^2+^ ion (Supplementary Figure S5), and ∼450 μM of trivalent Eu^3+^ (Supplementary Figure S6) and Gd^3+^ ions (Supplementary Figure S7) and the absorption spectral maximum position reverts back toward the free dye. The decrease in fluorescence intensity of SBE_7_βCD:C7H^+^ is plotted with the concentration of competitive binders as in Figure 10 and the inset shows that the effective fluorescence decreases at 400 μM concentration of competitive binders used in this study. The variation in the saturation concentrations of metal ions indicates that the trivalent metal ions compete very strongly with the dye toward the portals of SBE_7_βCD compared to the bivalent and monovalent metal ions. Overall, the competitive binding interaction of AHC and metal ions toward the SBE_7_βCD host follows the order: AHC > Eu^3+^or Gd^3+^> Ca^2+^> Na^+^. Though the fluorescence intensity of the SBE_7_βCD:C7 complex decreases upon the addition of AHC, the extent of the decrease is lesser, and a large concentration of AHC is required to reduce the intensity even by 20% (Supplementary Figure S8).

## Conclusion

In this study, we investigated the interaction of the SBE_7_βCD macrocyclic host with both the prototropic forms of coumarin dye to modulate the photophysical properties for possible biomolecular applications. Substantial enhancement in the fluorescence yield and lifetime of the dye in the presence of SBE_7_βCD indicates the confinement of both the forms of the dye (C7/C7H^+^) in the extended cavity of SBE_7_βCD which restricts the otherwise feasible non-radiative processes like TICT state formation and proton transfer interaction. The protonated C7H^+^ exhibits 3-fold higher binding interaction with SBE_7_βCD than the neutral C7 form which is corroborated by the electrostatic interaction of the cationic C7H^+^ with the sulfonate group at the portals and is seen in the large stabilization energy as well. C7 shows 0.4 units upward p*K*_a_ shift in the presence of SBE_7_βCD, whereas the shift is 0.5 units downward in the presence of parent βCD. The utility of the SBE_7_βCD:C7 complex for bioimaging applications has been demonstrated using confocal imaging of the *Drosophila* fly gut staining. The stimuli-responsive behavior of SBE_7_βCD:C7H^+^ was carried out in the presence of competitive binders such as amantadine hydrochloride and different metal ions to dissociate the complex and release the dye/drug, which is relevant for stimuli-responsive applications.

## Data Availability

The original contributions presented in the study are included in the article/Supplementary Material, further inquiries can be directed to the corresponding authors.
