# Supplementary material for: Supramolecular assembly of coumarin 7 with sulfobutylether-β-cyclodextrin for biomolecular applications
Source: Front Chem. 2023 Sep 5;11:1245518. doi: 10.3389/fchem.2023.1245518 (PMC10508339; doi:10.3389/fchem.2023.1245518)
Supplement: Supplementary file 1 [file DataSheet1.PDF]

Supplementary Material

Supramolecular Assembly of Coumarin 7 with Sulfobutylether-β-cyclodextrin for Biomolecular Applications

Gayathry T. C.,[†,#] Monika Gaur,[†,$,#] Lopamudra Mishra,[‡] Monalisa Mishra,[‡] Nilotpal Barooah,[†,$] Achikanath C. Bhasikuttan,[†,$,*] Jyotirmayee Mohanty[†,$,*]

[†]Radiation & Photochemistry Division, Bhabha Atomic Research Centre, Trombay, Mumbai 400085, INDIA

[$]Homi Bhabha National Institute, Training School Complex, Anushaktinagar, Mumbai, 400094, INDIA

[‡] Department of Life Science, National Institute of Technology Rourkela, Odisha 769008, India

Experimental method

Time-resolved fluorescence and anisotropy measurements

The time-resolved fluorescence measurements were carried out using a time-correlated single photon counting (TCSPC) set-up from Horiba Scientific (UK). In the present work, a 445 nm diode laser (100 ps, 1 MHz repetition rate) was used for the excitation of samples. A reconvolution procedure was used to analyze the observed decays, which could be satisfactorily fitted by mono- or biexponential decay functions. The fluorescence decays [I(t)] were analyzed in general as a sum of exponentials (Lakowicz, 2006; O'Connor, and Phillips,1984),

$$I(t) = \sum B_i \exp(-t/\tau_i) \qquad \text{(S1)}$$

where, B_i and τ_i are the pre-exponential factor and fluorescence lifetime for the ith component, respectively. Reduced chi-square (χ^2) values (within 1.00-1.20) and random distribution of the weighted residuals among data channels were used to judge the acceptance of the fits.

For anisotropy measurements, samples were excited with a vertically polarized excitation beam, and the vertically and horizontally polarized fluorescence decays were collected with a large spectral bandwidth of ~32 nm. Using these polarized fluorescence decays, the anisotropy decay function, r(t), was constructed as follows (Lakowicz, 2006; O'Connor, and Phillips,1984)

$$r(t) = \frac{I_V(t) - GI_H(t)}{I_V(t) + 2GI_H(t)} \qquad \text{(S2)}$$

I_V(t) and I_H(t) are the vertically and horizontally polarized decays, respectively, and G is the correction factor for the polarization bias of the detection setup. The G factor was determined independently by using a horizontally polarized excitation beam and measuring the two perpendicularly polarized fluorescence decays and the measurements were repeated at least three times.

Photostability Measurements

The stability of C7 and $SBE_7\beta CD$:C7 complex were evaluated/examined at room temperature. C7 and $SBE_7\beta CD$:C7 systems were kept at the ambient conditions and the photodegradation was monitored from their absorbance at the respective peak positions at different time intervals.

Bio-imaging

Adult flies (Oregon R) were maintained in a standard fly food and proper temperature for experimental set-up for control. Flies were dissected to take the guts away under the stereomicroscope using phosphate-buffered saline (PBS). The gut sample was collected and stored in 4 % paraformaldehyde (PFA) for 30 mins. For staining, the dissected gut sample was washed using 1x PBS, followed by washing with 1% PBST. Next, the guts were stained using 0.5 μM dye (in ethanol C-7 neutral dye) and kept up to 1 hour in the dark. The same concentration of the dye was used along with 1 mM $SBE_7\beta CD$ host for staining. After staining, guts were washed using 1xPBS and then mounted on a slide using 20% glycerol and coverslip. Images were taken in a time interval of 20 mins using a confocal microscope (Leica DMI8 Germany). Image analysis was done by using Image J 1.50i.

Supplementary Figures

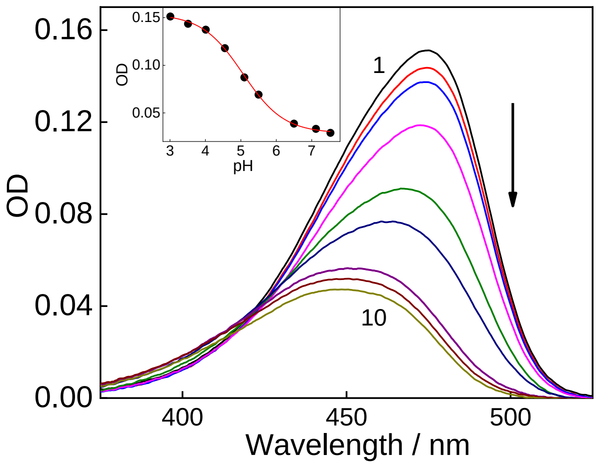

Figure S1. Absorption spectra of C7 with varying pH of the solutions. pH: 3.0 (1), 3.5 (2), 4.0 (3), 4.6 (4), 5.1 (5), 5.5 (6), 6.1 (7), 6.6 (8), 7.1 (9) and 7.5 (10). Inset shows the pK_a curve (variation of OD at 475 nm with pH of the solution) of C7.

Circular Dichroism study of C7 in the presence of SBE$_7$βCD

Induced circular dichroism (ICD) measurements on achiral guest molecules involved in a host−guest complexes involving cyclodextrins and their derivatives provide valuable information as these host molecules are chiral. The circular dichroism (CD) measurements have been carried out for different concentrations of C7 dye in the presence of 1 mM SBE$_7$βCD at both the pH conditions (Fig. S2). At pH 7, at lower concentration of C7, 462 nm absorption band shows a positive effect at the blue edge with a peak at ~425 nm and a negative effect at the red edge with a peak at ~475 nm (Fig. S2A), indicating an exciton splitting due to host-guest complex formation. With increasing concentration of C7 dye, the negative cotton effect disappears and a broad positive band appears with peak at ~460 nm which matches well with the absorption spectra of neutral C7 dye at pH 7. At higher dye concentration, most of the dyes remains in the uncomplexed form due to the lower binding constant and the CD spectrum corresponds to the absorption spectrum of free dye. However, at pH 3, C7 shows only positive cotton effects for the 469 nm band (Fig. S2B). The intensity of the band increases with increase in the cationic C7 concentration. Since the cationic form shows higher binding interaction than the neutral form, the positive cotton effects remain even at higher concentration of C7 at pH 3. A comparison of the CD spectra at pH 7 and pH 3 specifies the changes brought out in the induced chirality due to the geometrical distinction of the positioning of the dyes in the SBE$_7$βCD host.

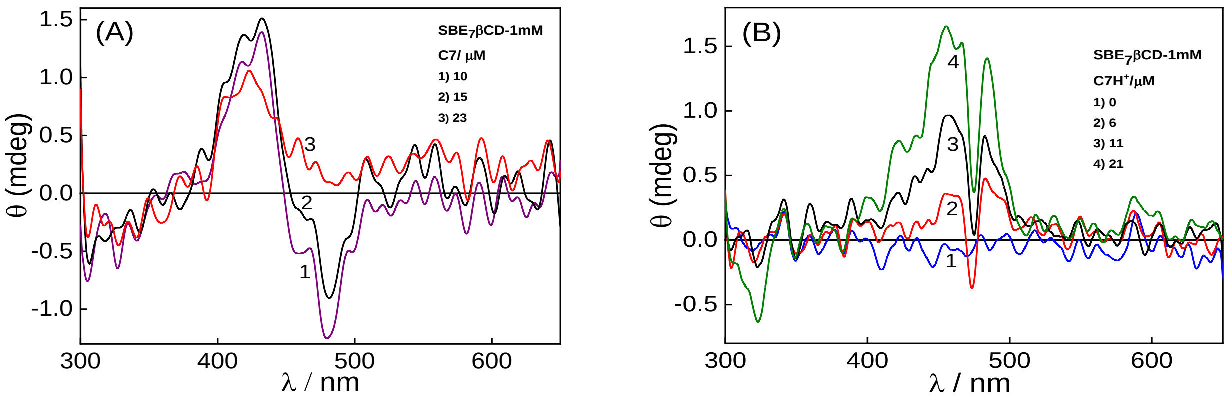

Figure S2. Circular dichroism spectra of different concentrations of C7 in the presence of 1mM SBE$_7$βCD at pH 7 (A) and pH 3 (B).

Comparison of photophysical properties of C7 with SBE$_7$βCD and βCD

Although SBE$_7$βCD contains the same hydrophobic cavity of βCD, the portals of SBE$_7$βCD is extended with the sulfobutyl ether groups (Jain et al., 2011; Loftsson and Brewster, 1996; Kale et al., 2005). The four secondary alcoholic groups in the wider portal and three alternate primary alcoholic groups in the narrow portal of βCD are substituted by sulfobutylether groups which facilitate the interaction with the cationic guest molecules (Shinde et al., 2015). Whereas, βCD shows moderate binding interaction towards

the neutral guest molecules through hydrogen bonding and hydrophobic interactions in the cavity (Khuran et al., 2019, Chem. Eur. J.; Kandoth et al., 2010). The changes at the portals account for the differential binding behavior of both the hosts towards the C7 dye. The protonated form $C7H^+$ shows three times higher binding interaction ((2.3±0.2) $\times 10^4$ M^{-1}) towards $SBE_7\beta CD$ than the neutral form ((8.1±0.8) $\times 10^3$ M^{-1}). The complexation through $SBE_7\beta CD$ favoured for the $C7H^+$ form due to the ion-dipole interactions, thus accounting for the difference in the binding constant values, whereas, in case of βCD, both the forms of C7 show similar binding interactions (~10^2 M^{-1}) (Chandrasekaran, et al., 2015) with a downward pK_a shift of ~0.5 unit (curve (c) of the inset of Fig. 7). In the presence of $SBE_7\beta CD$, the pK_a shift is about 0.4 unit in the upward direction. These shifts could be the combination of the partial binding of the dye in the hydrophobic βCD cavity which leads to the downward pK_a shift, whereas the portal binding, prominent with $SBE_7\beta CD$ leads to the upward pK_a shift and in effect the pK_a value is upwardly shifted about 0.9 unit by the SBE_7 arms. From the photophysical point of view, the present study as well as earlier studies suggest that $SBE_7\beta CD$ is a much better macrocyclic host for cationic dyes than βCD.

With amantadine hydrochloride (AHC)

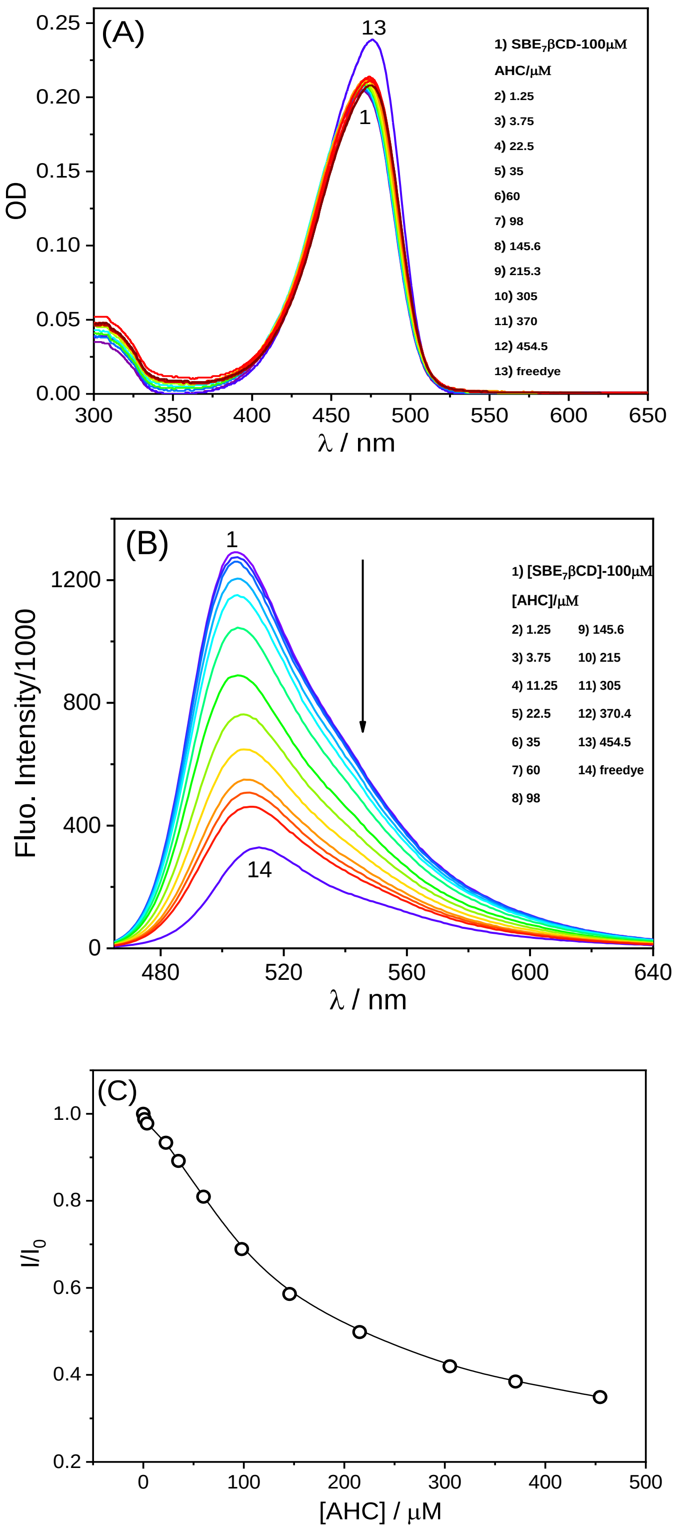

Figure S3. Absorption (A), fluorescence (B) spectra of SBE$_7$βCD (100 μM):C7$^+$ complex in the presence of different concentrations of AHC at pH 3. (C) Fluorescence intensity ratio of SBE$_7$βCD:C7$^+$ complex at 510 nm versus AHC concentration.

With Na⁺ ion

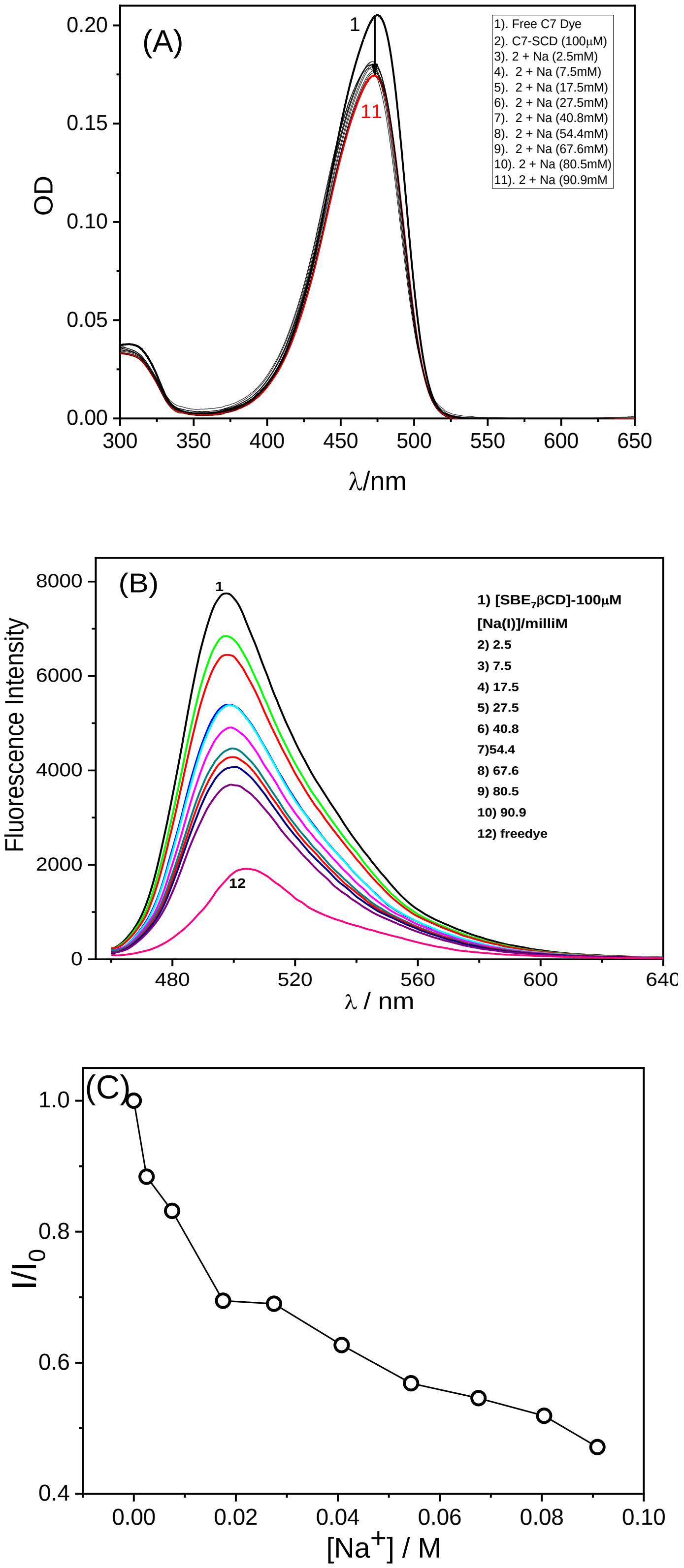

Figure S4. Absorption (A), fluorescence (B) spectra of SBE$_7$βCD (100 µM):C7H$^+$ complex in the presence of different concentrations of Na$^+$ ion at pH 3. (C) Fluorescence intensity ratio of SBE$_7$βCD:C7H$^+$ complex at 510 nm versus Na$^+$ ion concentration.

With Ca^{2+} ion

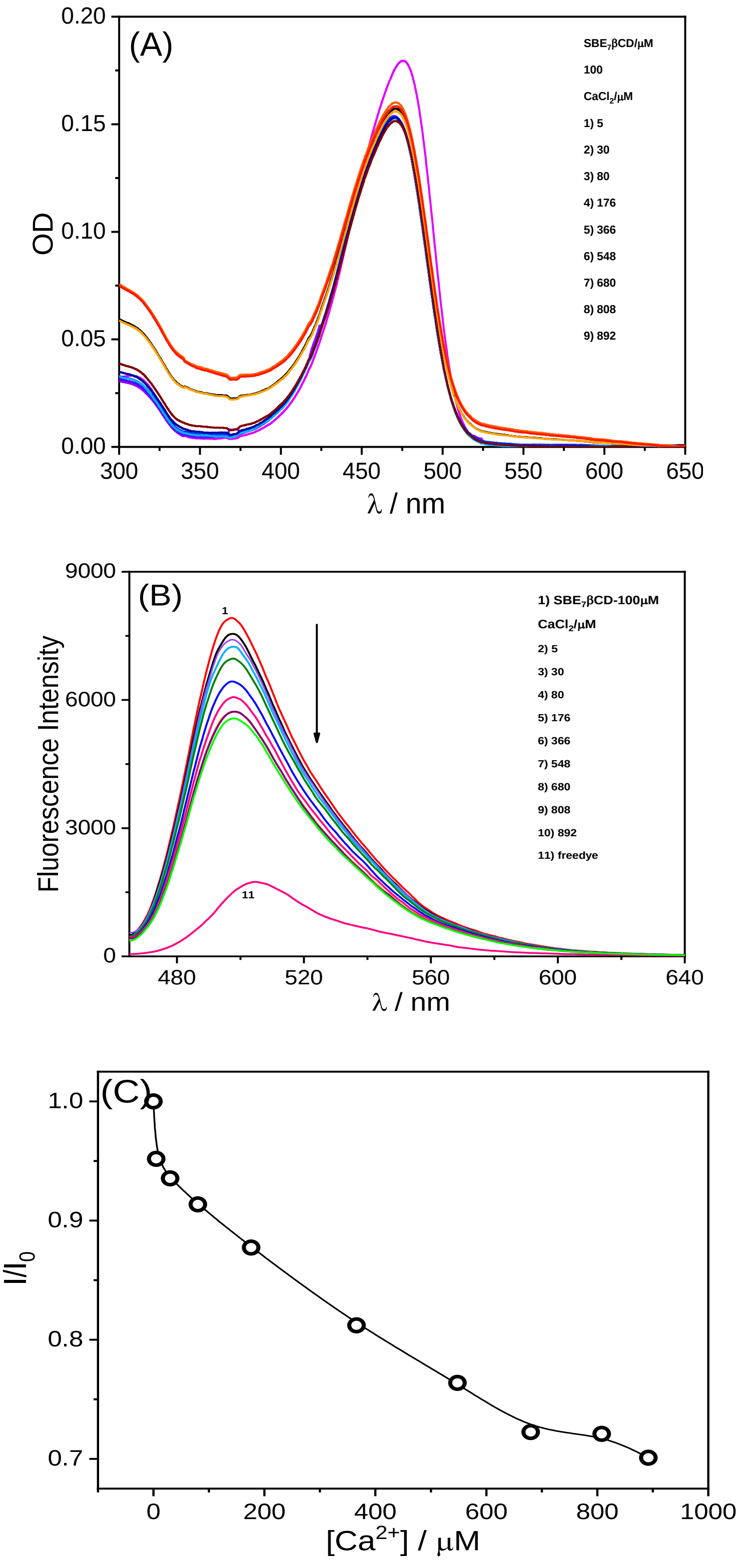

Figure S5. Absorption (A), fluorescence (B) spectra of SBE$_7$βCD (100 μM):C7H$^+$ complex in the presence of different concentrations of Ca^{2+} ion at pH 3. (C) Fluorescence intensity ratio of SBE$_7$βCD:C7H$^+$ complex at 510 nm versus Ca^{2+} ion concentration.

<u>With Eu^{3+} ion</u>

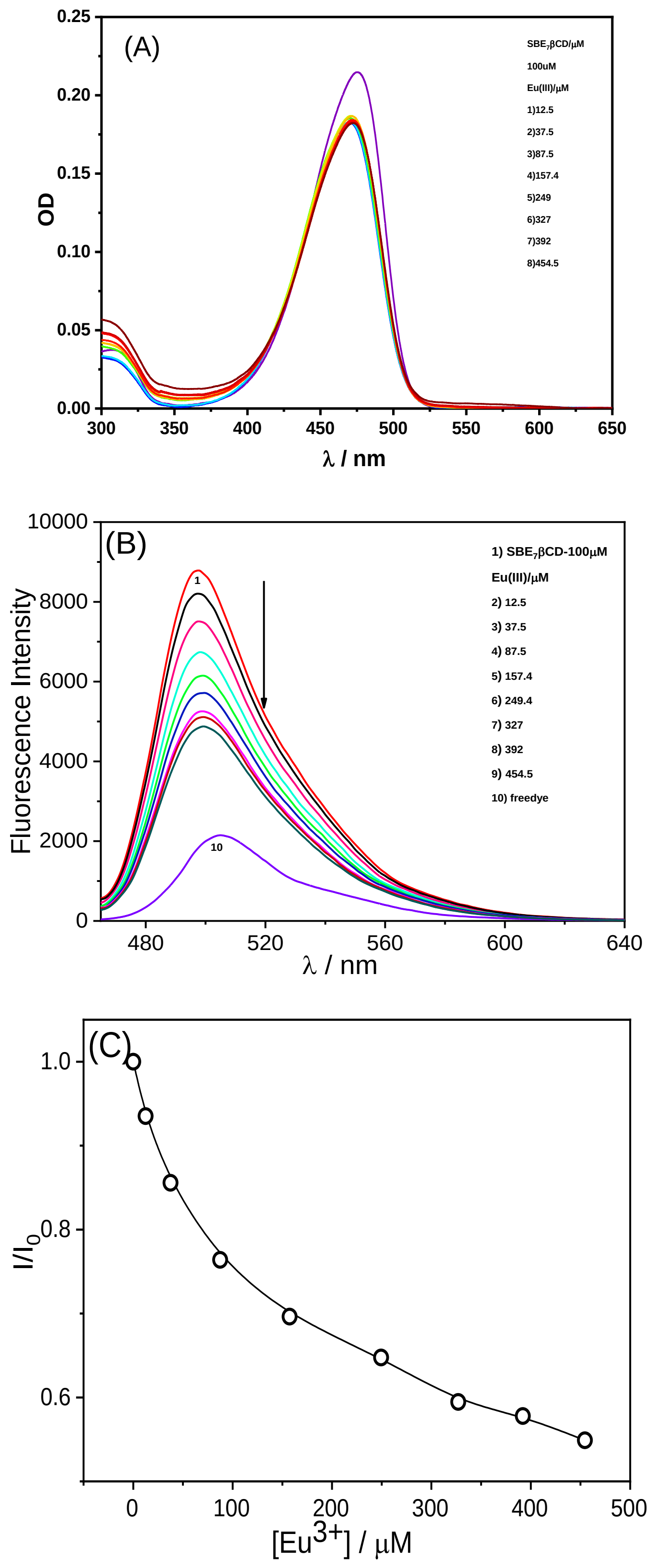

Figure S6. Absorption (A), fluorescence (B) spectra of $SBE_7\beta CD$ (100 μM):$C7H^+$ complex in the presence of different concentrations of Eu^{3+} ion at pH 3. (C) Fluorescence intensity ratio of $SBE_7\beta CD$:$C7H^+$ complex at 510 nm versus Eu^{3+} ion concentration.

With Gd^{3+} ion

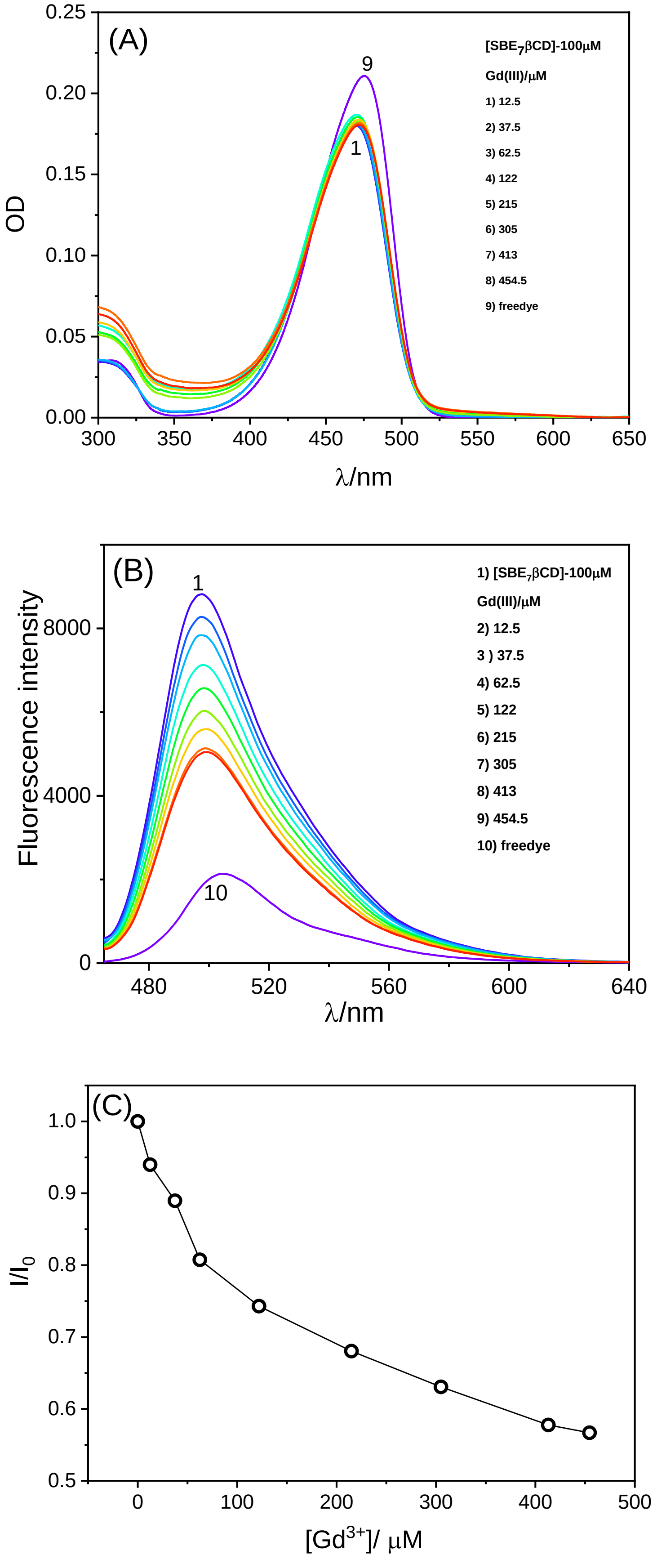

Figure S7. Absorption (A), fluorescence (B) spectra of $SBE_7\beta CD$ (100 μM):$C7H^+$ complex in the presence of different concentrations of Gd^{3+} ion at pH 3. (C) Fluorescence intensity ratio of $SBE_7\beta CD$:$C7H^+$ complex at 510 nm versus Gd^{3+} ion concentration.

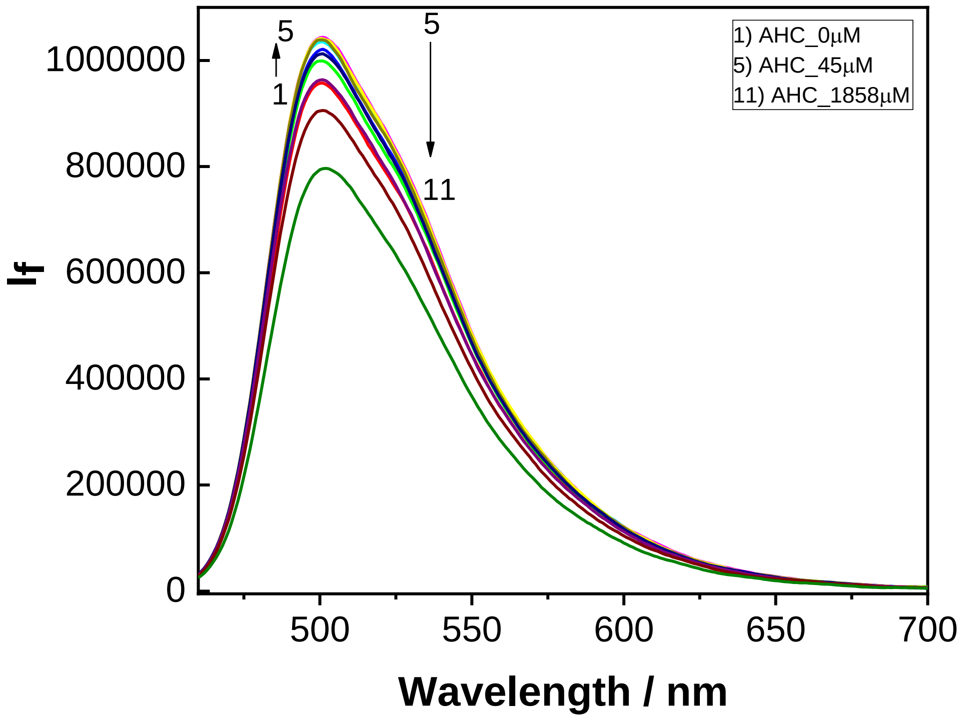

Figure S8. Fluorescence spectra of SBE$_7$βCD:C7 complex in the presence of different concentrations of AHC at pH 7.

<u>**References**</u>

Chandrasekaran, S., Sameena, Y., and Enoch I. V. (2015). Modulation of the interaction of Coumarin 7 with DNA by b-cyclodextrin. *J. Incl. Phenom. Macro. Chem.* 81, 225–236. Doi:10.1007/s10847-014-0451-1

Jain, A. S., Date, A. A., Pissurlenkar, R. R. S., Coutinho, E. C., and Nagarsenker, M. S. (2011). Sulfobutyl ether$_7$ β-cyclodextrin (SBE$_7$β-CD) carbamazepine complex: Preparation, characterization, molecular modeling, and evaluation of *in vivo* anti-epileptic activity. *AAPS PharmSciTech* 12, 1163–1175. Doi: 10.1208/s12249-011-9685-z

Kale, R., Saraf, M., and Tayade, P. (2005). Cyclodextrin complexes of valdecoxib: properties and anti-inflammatory activity in rat. *Eur. J. Pharm. Biopharm.* 60, 39–46. Doi: 10.1016/j.ejpb.2004.12.005

Kandoth, N., Dutta Choudhury, S., Mohanty, J., Bhasikuttan, A. C., and Pal, H. (2010). Inhibiting intramolecular electron transfer in flavin adenine dinucleotide by host-guest interaction: A fluorescence study. *J. Phys. Chem. B*, 114, 2617–2626. Doi:10.1021/jp909842z

Khurana, R., Agarwalla, S., Sridhar, G., Barooah, N., Bhasikuttan, A. C., and Mohanty, J. (2018). Ultra-bright rhodamines with sulfobutylether-β-cyclodextrin: A viable supramolecular dye laser in aqueous medium. *ChemPhysChem* 19, 2349□2356. Doi:10.1002/cphc.201800373

Khurana, R., Barooah, N., Bhasikuttan, A. C., and Mohanty, J. (2019). Supramolecular assembly induced emission of thiazole orange with sulfobutylether β-cyclodextrin: A stimuli-responsive fluorescence sensor for tyramine. *ChemPhysChem* 20, 2498–2505. Doi:10.1002/cphc.201900656

Lakowicz, J. R. (2006). Principles of Fluorescence Spectroscopy. 3rd ed.; Springer: New York.

Loftsson, T., and Brewster, M. E. (1996). Pharmaceutical applications of cyclodextrins. 1. Drug solubilization and stabilization. *J. Pharm. Sci.* 85, 1017–1025. Doi: 10.1021/js950534b

O'Connor, D. V., and Phillips, D. (1984). Time correlated single photon counting. Academic Press: New York.

Shinde, M. N., Bhasikuttan, A. C., and Mohanty, J. (2015). Contrasting recognition behavior of β-cyclodextrin and its sulfobutylether derivative towards 4',6-Diamidino-2-phenylindole (DAPI). *ChemPhysChem* 16, 3425-3432. Doi: 10.1002/cphc.201500638
